# Nitrogen oxides (NO and NO_2_) pollution in the Accra metropolis: Spatiotemporal patterns and the role of meteorology

**DOI:** 10.1016/j.scitotenv.2021.149931

**Published:** 2021-08-27

**Authors:** Jiayuan Wang, Abosede Sarah Alli, Sierra Clark, Allison Hughes, Majid Ezzati, Andrew Beddows, Jose Vallarino, James Nimo, Josephine Bedford-Moses, Solomon Baah, George Owusu, Ernest Agyemang, Frank Kelly, Benjamin Barratt, Sean Beevers, Samuel Agyei-Mensah, Jill Baumgartner, Michael Brauer, Raphael E Arku

**Affiliations:** 1 Department of Environmental Health Sciences, School of Public Health and Health Sciences, University of Massachusetts, Amherst, USA; 2 Department of Epidemiology and Biostatistics, School of Public Health, Imperial College, London, UK; 3 Department of Physics, University of Ghana, Legon, Ghana; 4 MRC Centre for Environment and Health, Imperial College London, London, UK; 5 Abdul Latif Jameel Institute for Disease and Emergency Analytics, Imperial College London, London, UK; 6 Regional Institute for Population Studies, University of Ghana, Accra, Ghana; 7 NIHR HPRU in Environmental Exposures and Health, Imperial College London, UK; 8 Harvard T.H. Chan School of Public Health, Boston, MA, USA; 9 Insitute of Statistical, Social and Economic Research, University of Ghana, Legon, Ghana; 10 Department of Geography and Resource Development, University of Ghana, Legon, Ghana; 11 Institute for Health and Social Policy, McGill University, Montreal, Canada; 12 Department of Epidemiology, Biostatistics, and Occupational Health, McGill University, Montreal, Canada; 13 School of Population and Public Health, The University of British Columbia, Vancouver, Canada

**Keywords:** Nitrogen oxides, traffic, sub-Saharan Africa, Ghana, Harmattan, meteorology, mixing layer depth, incident solar radiation, COVID-19

## Abstract

Economic and urban development in sub-Saharan Africa (SSA) may be shifting the dominant air pollution sources in cities from biomass to road traffic. Considered as a marker for traffic-related air pollution in cities, we conducted a city-wide measurement of NO_x_ levels in the Accra Metropolis and examined their spatiotemporal patterns in relation to land use and meteorological factors. Between April 2019 to June 2020, we collected weekly integrated NO_x_ (n=428) and NO_2_ (n=472) samples at 10 fixed (year-long) and 124 rotating (week-long) sites. Data from the same time of year were compared to a previous study (2006) to assess changes in NO_2_ concentrations. NO and NO_2_ concentrations were highest in commercial/business/industrial (66 and 76 μg/m^3^, respectively) and high-density residential areas (47 and 59 μg/m^3^, respectively), compared with peri-urban locations. We observed annual means of 68 and 70 μg/m^3^ for NO and NO_2_, and a clear seasonal variation, with the mean NO_2_ of 63 μg/m^3^ (non-Harmattan) increased by 25-56% to 87 μg/m^3^ (Harmattan) across different site types. The NO_2_/NO_x_ ratio was also elevated by 19-28%. Both NO and NO_2_ levels were associated with indicators of road traffic emissions (e.g. distance to major roads), but not with community biomass use (e.g. wood and charcoal). We found strong correlations between both NO_2_ and NO_2_/NO_x_ and mixing layer depth, incident solar radiation and water vapor mixing ratio. These findings represent an increase of 25 – 180% when compared to a small study conducted in two high-density residential neighborhoods in Accra in 2006. Road traffic may be replacing community biomass use (major source of fine particulate matter) as the prominent source of air pollution in Accra, with policy implication for growing cities in SSA.

## 1 Introduction

Air pollution is a major environmental health threat globally, and both the amount and impact are estimated to be highest in Asia and Africa.^1^ In Sub-Saharan Africa (SSA), the world’s fastest urbanizing region,^2^ the combination of urban population and economic growth may be raising air pollution levels from diverse sources, particularly combustion related sources.^3–5^ Motorization in terms of the volume, distance travelled, and activity is rapidly growing in SSA’s sprawling cities along with persistence of older, more polluting imported vehicles in the fleet.^6,7^ For example, Ghana’s population grew by ~70%, while registered vehicles (mean age = 14 years^8^) increased more than eight times since 2000.^9^ Excessive traffic congestion undermines economic productivity by increasing commuting time and costs and road traffic can be a major source of particulate matter (PM) and nitrogen dioxide (NO_2_) pollution in cities.^10,11^ Other combustion sources of air pollution in SSA cities include biomass use, an important source of particulate matter pollution, and diesel generators for household and commercial activities, informal industries, and household trash burning^4,5^. Together with traffic, these sources influence the outdoor air pollutant mixture in SSA cities.^12^ But similar to developed countries, road traffic emissions may now be the dominant source of urban air pollution in SSA cities amid the increasing expansion, motorization, and downward trend in primary biomass use ^10,11,13^.

As frequently used markers for traffic-related air pollution,^14–16^ outdoor NO_2_ and other nitrogen oxides (NO_x_) are important pollutants in most American, European and Asian cities. Concerns over their adverse health impacts and contributions to secondary PM and ozone (O_3_) formation^17,18^ have resulted in national regulations and international guidelines to minimize population exposures.^1,18^ Besides traffic emissions, sustained household use of biomass fuels in SSA is considered an important source of NO_2_ pollution in cities.^5^ As SSA rapidly urbanizes, cities face “double threat” of NO_x_ pollution: although declining, household biomass use remains substantial, while the influence of road traffic is rising. Consequently, there is a likely shift in emission sources from traditional biomass (PM dominant) toward “modern” road-traffic (NO_x_ dominant), similar to cities in high-income countries.^12,19^ In addition to local emissions from combustion sources, seasonal changes in regional meteorology have significant influences on local air quality within the West African sub-region.^20^ Specifically, the dry, dusty Harmattan period (usually around November-February) characterized by north-easterly trade winds from the Sahara Desert worsens air quality through transboundary transport of mineral dusts and smoke from biomass burning. ^12,21–25^ Conversely, the wet monsoon season (around April-October) improves air quality across the subregion due to stronger convection and wet removal.^20,22,25,26^ Thus, systematic and city-wide NO_x_ data are needed to improve our understanding of air pollution and ensure effective urban air quality management in SSA cities that are in economic transition from low to middle/high income status, and accompanied by a transition to road traffic as a dominant source of urban air pollution.^5,19,27^

In a large city-wide campaign, we conducted a yearlong field measurement of NO_2_ and NO_x_ concentrations at 134 locations within the Greater Accra Metropolitan Area (GAMA), one of the fastest growing metropolises in the West African sub-region. This paper describes the space-time variation of the measured NO_2_ and NO_x_ concentrations in relation to diverse land use factors across communities in the GAMA. We further assess the role of meteorology and seasonality on NO_2_ and NO_x_ concentrations.

## 2 Methodology

### 2.1 Study location

This study took place in the GAMA, the most urbanized area of Ghana and hosts more than 60% of the country’s registered vehicles.^9^ Located along the Atlantic coast, the GAMA covers about 1500 km^2^ with the population of ~ 5 million,^28^ growing at ~3%^29^. It contains the old Accra Metropolitan Area (AMA) as its core, the fast-growing port and industrial city of Tema Metropolitan Area (TMA) to the east, and the surrounding peri-urban municipalities to the north east and north west. The central business district of Accra experiences an estimated one million passenger trips per day from Trotros (old imported minibuses used primarily for public transport) and taxis;^30^ a number that is expected to rise with urban sprawl. Like the rest of the country, the GAMA lies in dry coastal equatorial climatic zone with wet (April to October) and dry dusty Harmattan seasons (November to February).^20,31^ The average monthly temperature ranges from 25 to 33°C (77 – 91 F) while average daily humidity is at about 83%.

### 2.2 Study design

The study was nested within a large multi-country and multi-city “Pathways to Equitable Healthy Cities” study (http://equitablehealthycities.org/), which aims to improve population health, enhance health equity and ensure environmental sustainability in six study cities around the world. Detailed description of the full campaign protocol, which was part of the larger environmental monitoring campaign in the “Pathways to Equitable Healthy Cities” study, can be found elsewhere.^32^ Briefly, we collected weekly pairs of integrated NO_x_ and NO_2_ samples at a combination of ‘fixed’ (year-long; n=10) and ‘rotating’ (week-long; n=124) sites to capture both the temporal (annual, seasonal, and weekly) and spatial variability across the GAMA. The location of the rotating sites were chosen using a stratified random approach based on population and land cover data from the World Bank^33^ to capture various land-use and socioeconomic factors: traffic areas, high-, and low-density residential neighborhoods, and peri-urban sites. The 10 fixed sites were selected deliberately to represent diverse geography, population density, road-traffic and road-networks, and neighborhood biomass fuel use based on 2010 national census. Relative to the entire GAMA, the sampling sites were over-represented in the more densely populated AMA (n= 51: 6 fixed and 45 rotating sites). Measurements took place between July 2019 and June 2020, following a one-month pilot study in April 2019. In each measurement week, we collected data simultaneously at the 10 fixed (year-long) sites along with five rotating (week-long) sites throughout the campaign. Given regular traffic congestion in the city, the five rotating sites for a particular measurement week were chosen in proximity to each other for easy access. A duplicate (side-by-side) sample and a field blank were collected each week at one (20%) of the five rotating sites throughout the campaign. Due to the COVID-19 pandemic, our field campaign was suspended between March and early May 2020, partly because Accra implemented partial lockdown and partly because our field team had to self-isolate through contact tracing. During the lockdown, individuals were directed to stay at home except for essential items (e.g. food, medicine, and water). Travel to and within Accra was also suspended (except for essential goods and services), while passenger vehicles (e.g. trotros) had to reduce the number passengers per trip to observe social distancing. The field campaign resumed shortly after, allowing us to glean information about the impact of the lockdown on local emissions in the city. In summary, we collected 281 NO_2_ and 251 NO_x_ weekly samples in the pre-COVID-19 lockdown, 19 pairs during COVID-19 lockdown, and 50 pairs in the post-COVID-19 lockdown periods.

### 2.3 NO_x_ and NO_2_ measurements

Pairs of weekly integrated ambient NO_x_ and NO_2_ samples were collected using Ogawa passive samplers (Ogawa & Co., Inc., USA), which captured NO_x_ and NO_2_ concentrations on pre-coated collection pads. The samplers were deployed on metal poles at a height of ~4 m above ground and covered by an opaque plastic container that served as a weather shield. After collection, the filters were sealed in vials and refrigerated at 4 °C prior to its cold courier to the University of Massachusetts Amherst for laboratory analysis. We followed Ogawa’s analytical protocol by first extracting the samples in Milli-Q water, and then added color reagent (sulfanilamide [99%, Sigma, USA] and N-(1-Naphthyl)-ethylenediamine dihydrochloride [99%, Sigma, USA]) and allowed to equilibrate at room temperature for 20 minutes. The developed color was measured at 545 nm wavelength by a spectrophotometer (SpectraMax M2e, USA). Each sample was measured three times to ensure precision and the average of all three was used for calculating the final concentrations. Using the total sampling time, concentrations of NO_x_ and NO_2_ were then calculated by linear calibration line, created from nitrite standard solution (Thermo Fisher, USA) and corrected for temperature and relative humidity measured at six of the ten fixed site locations throughout the measurement campaign. For easy comparison of our NO_x_ and NO_2_ levels with other studies and international health guidelines, we report all results in the unit of μg/m^3^.

### 2.4 Data management and statistical analysis

The final data used in this analysis were blank corrected. We calculated a limit of detection (LOD) separately for NO_x_ and NO_2_ as three times the standard deviation (SD) of their field blanks. The LODs were 0.07 and 0.02 μg/m^3^ for NO_x_ and NO_2_, respectively. The duplicate samples were strongly correlated (*R^2^* = 0.98 for NO_x_ and 0.95 for NO_2_; Figure S1). Consequently, samples at duplicate sites were averaged to provide a single estimate at these sites. Though we could not co-locate against a reference monitor for comparison in Ghana’s climatic conditions, the Ogawa samplers have been well-characterized in field settings with good agreements^34,35^, including in similar setting as our study ^36^.

To assess variations in community level concentrations by land use factors, we categorized each individual monitoring station into one of four land-use categories: (i) commercial/business/industrial (CBI) – areas with commercial ventures, industrial activities or government offices, which are often along major motorways or highways; (ii) high-density residential (HD) – informal or formal densely populated residential neighborhoods with narrow paved or unpaved roads, low socioeconomic status (SES) and high biomass use; (iii) low-density residential (LD) – formal, sparsely populated, high SES, low biomass use residential communities with medium to wide roads; and (iv) peri-urban background (UB) – areas with high green space with little or no direct influence from traffic and biomass smoke. We describe the spatial patterns of the measured NO_x_ and NO_2_ concentrations by this land-use classification.

Because the weekly samples from the rotating sites were collected in groups of five in different parts of the city across different months and seasons, we conducted temporal/seasonal adjustment on the concentrations measured at the rotating sites in order to remove temporal variations and allow comparison across sites. This approach also allowed us to obtain seasonal and annual mean equivalents for all sites. For each sampling week, a temporal factor (TF) for that week was calculated as the ratio of the weekly mean value to the annual mean at all fixed sites.

Concentrations from the rotating sites were adjusted for ‘time trends’ by dividing the samples by the TF for that week.^37^ The adjusted concentration ${\left(C_{i}^{\mathrm{Rotating}\;\mathrm{Site}}\right)}_{j}^{*}$ of the *i* rotating site for the *j* week was calculated as:

(1) $$TF={\left(C^{\mathrm{Fixed}\;\mathrm{Site}}\right)}_{j}/\bar{(C^{\mathrm{Fixed}\;\mathrm{Site}}})$$

(2) $${\left(C_{i}^{\mathrm{Rotating}\;\mathrm{Site}}\right)}_{j}^{*}={\left(C_{i}^{\mathrm{Rotating}\;\mathrm{Site}}\right)}_{j}/TF$$

where (*C^Fixed Site^*)_*j*_ is the average NO_2_ or NO_x_ concentration at all fixed sites in the *j* measurement week, $\bar{\left(C^{\mathrm{Fixed}\;\mathrm{Site}}\right)}$ is the mean annual concentration at all fixed sites, and (*C_i_^Rotating Site^*)_*j*_ is the NO_x_ or NO_2_ concentration measured at the *i* rotating site in the corresponding *j* measurement week. The median (interquartile range) of the TFs were 1.0 (0.9 – 1.2) for NO_x_ and 1.0 (0.8 – 1.1) for NO_2_ (Figure S2).

We used the seasonally adjusted data from the rotating sites to assess the spatial patterns across the GAMA, and evaluated by the land use characteristics described above. The year-long data from the fixed sites were used to examine annual mean concentrations and seasonal trend in terms of the Harmattan (the dry and dusty northeasterly trade wind from the Sahara Desert, November to February^20^) and non-Harmattan periods. We also tested the impact of local changes in regional meteorology and transboundary pollution (e.g. smoke from biomass burning transported along with Sahara dusts during the Harmattan) on NO_x_ concentrations in the GAMA by evaluating mixing layer depth, incident solar radiation and water vapor mixing ratio throughout the campaign period using the Global Data Assimilation System (GDAS1) data downloaded from the National Oceanic and Atmospheric Administration (NOAA) (ftp://arlftp.arlhq.noaa.gov/pub/archives/gdas1), and output by the Hybrid Single-Particle Lagrangian Integrated Trajectory (HYSPLIT) 4 model (https://www.arl.noaa.gov/hysplit/hysplit/), which contains information on air parcel trajectories, transport, and dispersion.^38^ Further, we compared time equivalent data with a 2006 study to assess changes in NO_2_ levels within the AMA over the last decade.^19^

Because NO_x_ is comprised primarily of nitric oxide (NO) and NO_2_, we operationally define NO_x_ as NO + NO_2_. Our final results are presented as NO (i.e. NO_x_ – NO_2_) and NO_2_. We used an alpha of 0.05 as cut-off for statistical significance. Data analyses, visualizations, and all summary statistics on the spatial and temporal trends were performed in RStudio (R version 3.6.1).

## 3 Results

**Figure 1:**
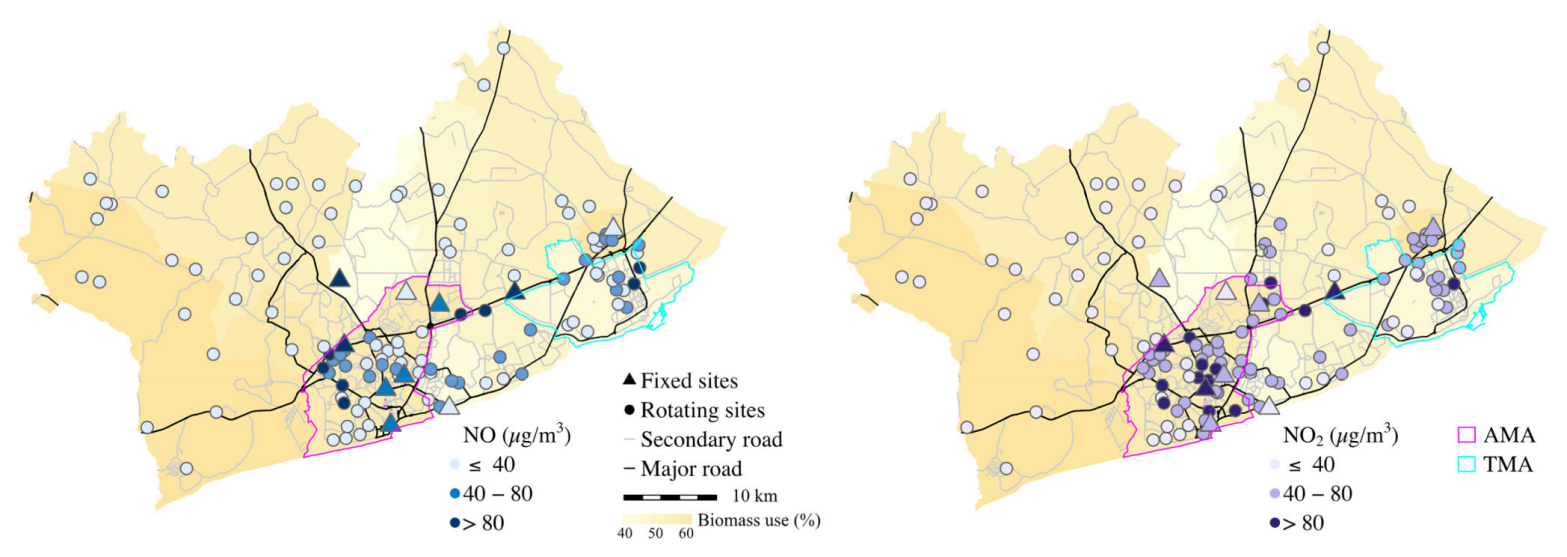
Location of the sampling sites and annual concentrations of (A) NO and (B) NO_2_ across the GAMA. The colors of NO_2_ concentrations indicate comparison to the WHO annual guideline of 40 μg/m^3^. The concentrations at the fixed sites represent annual mean values, and the rotating sites represent season-adjusted mean values (i.e. an estimated annual means). Major and secondary road network were from OpenStreetMap (downloaded in 2019). Biomass use data and the GAMA boundaries were from Ghana Statistical Service (2010 Census).

We collected a total of 428 (2,996 site-days) and 472 (3,318 site-days) weekly integrated NO and NO_2_ samples, respectively, at 10 fixed (year-long) and 124 rotating (week-long) sites. The location of the sampling sites across the GAMA and their respective annual NO and NO_2_ concentrations (in comparison to WHO annual guideline of 40 μg/m^3^) are shown in Figure 1.

### 3.1 Spatial patterns

**Figure 2:**
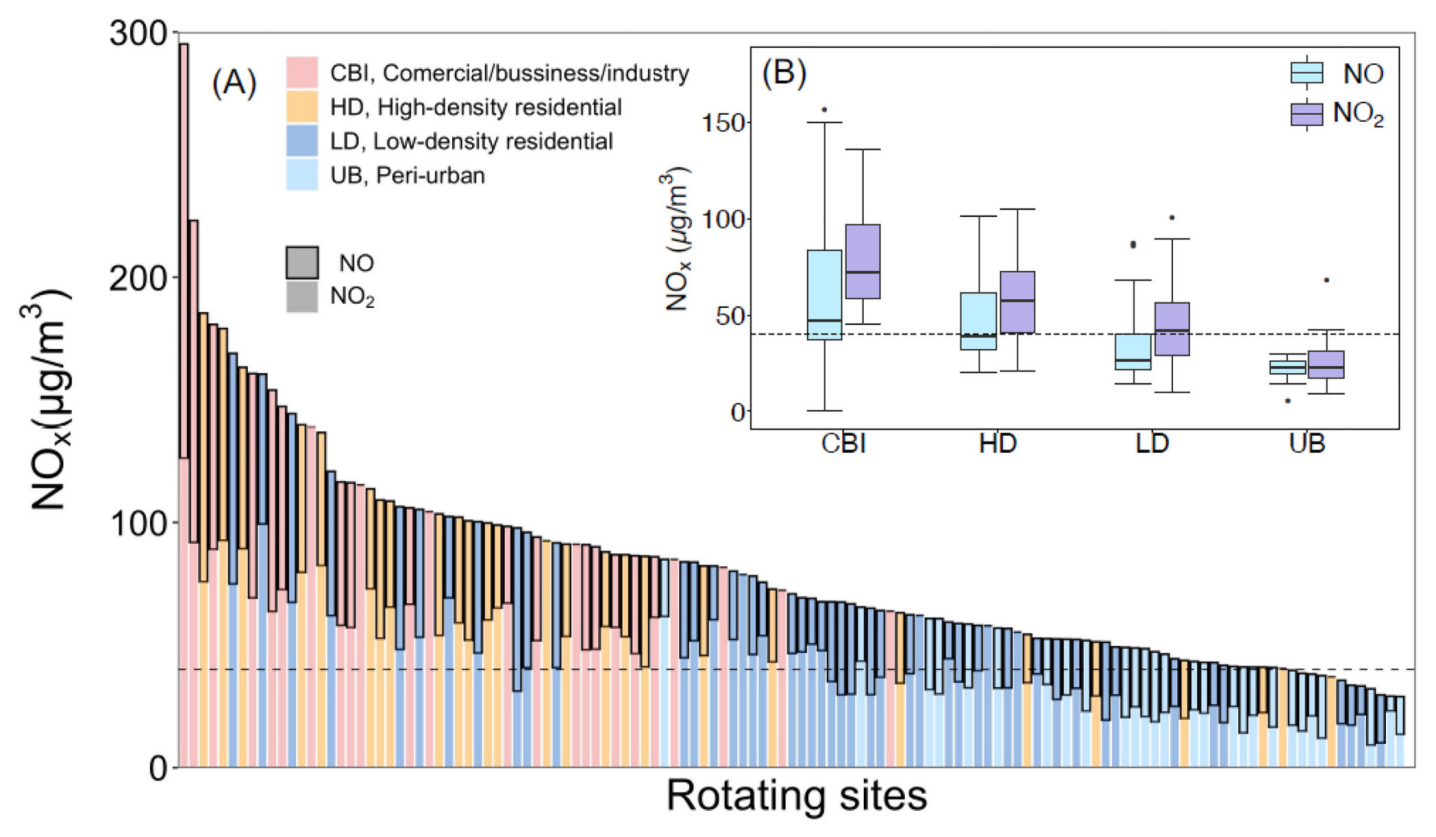
Distribution of NO_x_ (NO_x_ = NO + NO_2_) concentrations by site-type. (A) Site-specific values at each rotating site. (B) Median and interquartile range values. The dash lines are the WHO guideline for annual NO_2_ concentrations of 40 ug/m^3^.

The season-adjusted mean NO and NO_2_ concentrations across all the rotating sites were 39 (range: 6 – 156) μg/m^3^ and 50 (range: 9 – 136) μg/m^3^, respectively (Table 1). Both NO and NO_2_ concentrations varied substantially by neighborhood characteristics and land-use features. Levels were highest in CBI areas (mean NO = 66 μg/m^3^ and NO_2_ = 80 μg/m^3^), which are dominated by heavy vehicular traffic, followed by sites in HD residential neighborhoods (mean NO = 47 μg/m^3^ and NO_2_ = 59 μg/m^3^) with relatively less traffic (Figure 2). Concentrations in LD residential neighborhoods were lower compared to HD and CBI areas, but were significantly higher than UB locations, which registered the lowest values (mean NO and NO_2_: 34 and 45 vs 27 and 24 μg/m^3^, respectively) (Figure 2B). Pairwise analysis of variance revealed significant differences in the mean concentrations of both NO and NO_2_ across each category of site-type (*p* < 0.05). When compared by degree of urbanization, the mean NO and NO_2_ concentrations were highest in communities in the most densely populated AMA (51 μg/m^3^ and 69 μg/m^3^), followed by the TMA (42 μg/m^3^ and 50 μg/m^3^) where the port is located, and lowest in the other adjoining municipalities combined (32 μg/m^3^ and 37 μg/m^3^) (Table 1). Sites near major and medium roads registered significantly higher overall mean concentrations than sites near minor roads and alleys (NO: 88 vs 34 μg/m^3^, *p* < 0.001; and NO_2_: 89 vs 45 μg/m^3^, *p* < 0.001).

### 3.2 Temporal patterns

#### 3.2.1 Annual levels

The overall mean annual NO concentration across the ten year-long (fixed) sites was 63 μg/m^3^ and site-specific mean annual concentrations ranged from 20 μg/m^3^ at a UB site to 118 μg/m^3^ at CBI sites; NO_2_ followed same pattern with mean annual of 68 μg/m^3^ and ranged between 28 μg/m^3^ and 98 μg/m^3^ at different sites. Site-specific mean annual NO_2_ concentrations at all fixed sites, and 78% (n = 279) of the 350 total fixed site samples (except UB), exceeded the 40 μg/m^3^ WHO annual guideline. The mean annual NO levels in HD and LD residential communities were similar (42 vs 45 μg/m^3^), but NO_2_ concentrations were higher in HD than in LD neighborhoods (71 vs 56 μg/m^3^; *p* < 0.001) (Table 2). Unlike the data from rotating (week-long) sites, NO/NO_x_ ratios at the year-long sites, which were overrepresented in the more populated areas of the GAMA, showed varied spatial patterns by site-type: they were highest at CBI (ratio: 0.53) sites, similar at LD and UB areas (0.42), and lowest at HD sites (0.37) (Table 2). In general, we observed a drastic increase in the NO/NO_x_ ratios with increasing NO_x_ at CBI sites, indicative of fresh and direct emissions of NO from traffic (Figure S3).

#### 3.2.2 Seasonal patterns

**Figure 3:**
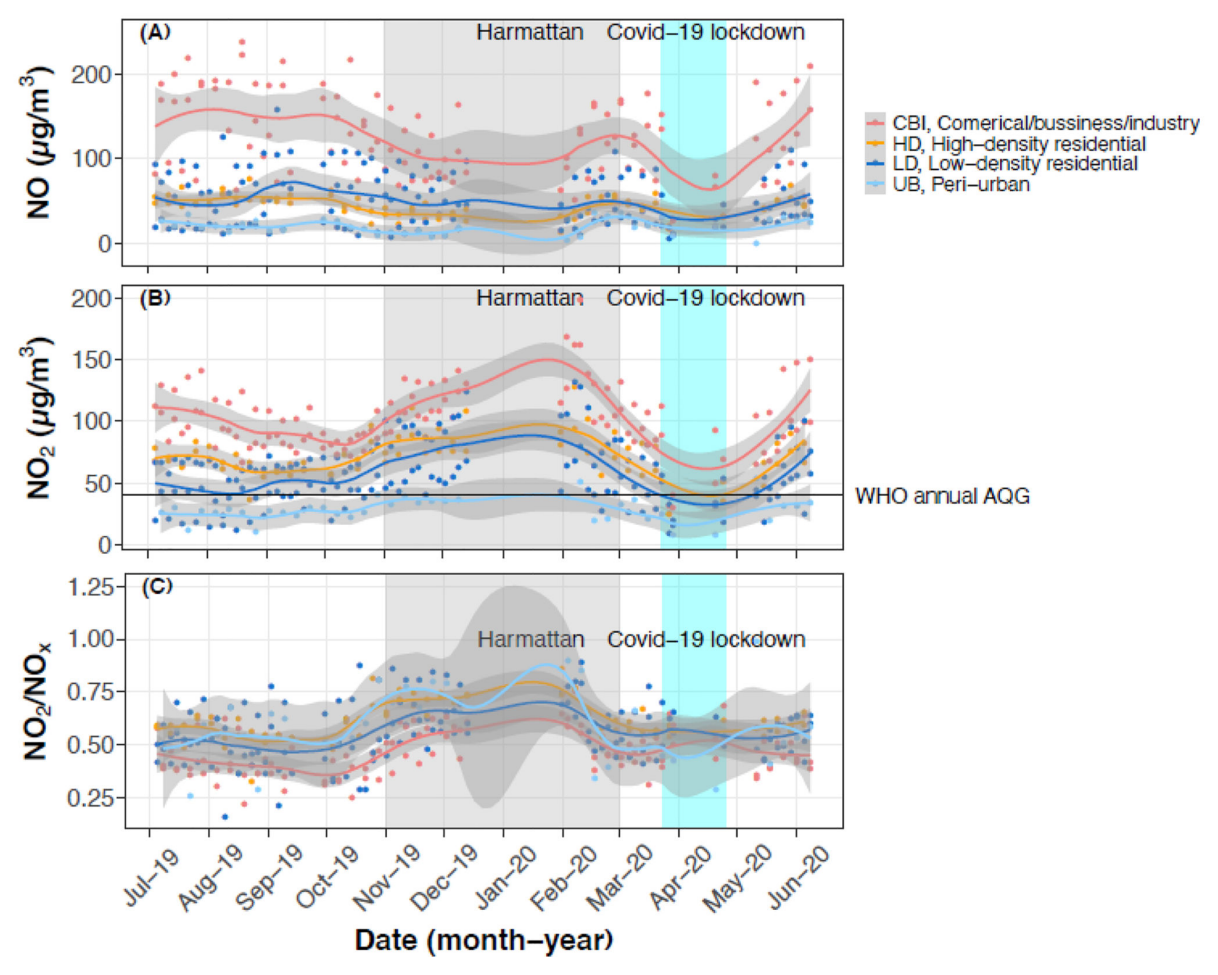
Time series of (A) NO, (B) NO_2_ concentrations, and (C) NO_2_/NO_x_ ratios at the fixed (year-long) measurement sites and grouped by site-type. The pilot data was excluded in the figure. The points represent individual weekly integrated samples and the lines represent the smoothed trend (method = “loess”) with their standard errors. The black line in (B) represents the WHO guideline for annual NO_2_ concentrations of 40 ug/m^3^. * The field campaign was briefly suspended over the Christmas holidays and also for mid-campaign QA/QC as described in our protocol,^32^ which likely biased our annual mean results downward. ** There was missing data due to COVID-19 lockdown of Accra between March and April 2020 as well as mandatory quarantine for the field team through contact tracing.

The year-long data from the ten fixed sites demonstrate clear seasonal patterns, with overall decreases in NO compared to notable increases in NO_2_ during the Harmattan period (Nov 2019 - Feb 2020) (Figure S4). We observed similar (and clearer) pattern when data from both fixed and rotating sites were combined (Figure S5). Although COVID-19 lockdown resulted in fewer Harmattan samples than initially planned, we still obtained enough data to gain insight into the impact of the dusty Harmattan on the measured levels. The mean NO concentration showed a 10% drop during Harmattan compared to non-Harmattan (59 vs. 66 μg/m^3^), while NO_2_ increased significantly by 45% (NO_2_: 87 vs. 60 μg/m^3^), and was more than double the WHO guideline in the Harmattan alone (Figure S4 and Table 2). Seasonality in both NO and NO_2_ concentrations persisted across all site types. While NO decreased slightly, NO_2_ increased by 35-56% during the Harmattan at CBI, HD, and LD areas. Interestingly, NO_2_ increases were also seen at the UB site (by ~25%) during the Harmattan, but with corresponding decreases in NO (Figure 3, Figure S5, Table 2), suggesting a regional/transboundary (meteorologic) impact rather than increases in local emissions. Similarly, the NO_2_/NO_x_ ratios at all sites increased notably by 18-27% during Harmattan (Figure 3C and Table 2), with UB having the highest change (0.69 vs 0.54), indicative of the enhancement in local NO_2_ production. Overall, equivalent annual and seasonal (Harmattan vs non-Harmattan) mean estimates at all monitoring sites demonstrate strong interplay between site-type (source influence) and season influence (Figure S5).

#### 3.2.3 Change in NO_2_ concentration in AMA since 2006

In 2006 weekly measurement at 26 sites in two HD neighborhoods in the AMA, Arku et al (2008) reported mean NO_2_ concentration of 21 ppb (39 μg/m^3^), ranging between 20 and 22 μg/m^3^ at small roads/alleys to 66 μg/m^3^ near major roads,^19^ For the same time period in similar neighborhoods in this present 2019/2020 study, the mean NO_2_ levels ranged between 53 and 101 μg/m^3^; this represents 53 – 152 % increase over the 2006 levels.

#### 3.2.3 Effects of the harmattan

**Figure 4:**
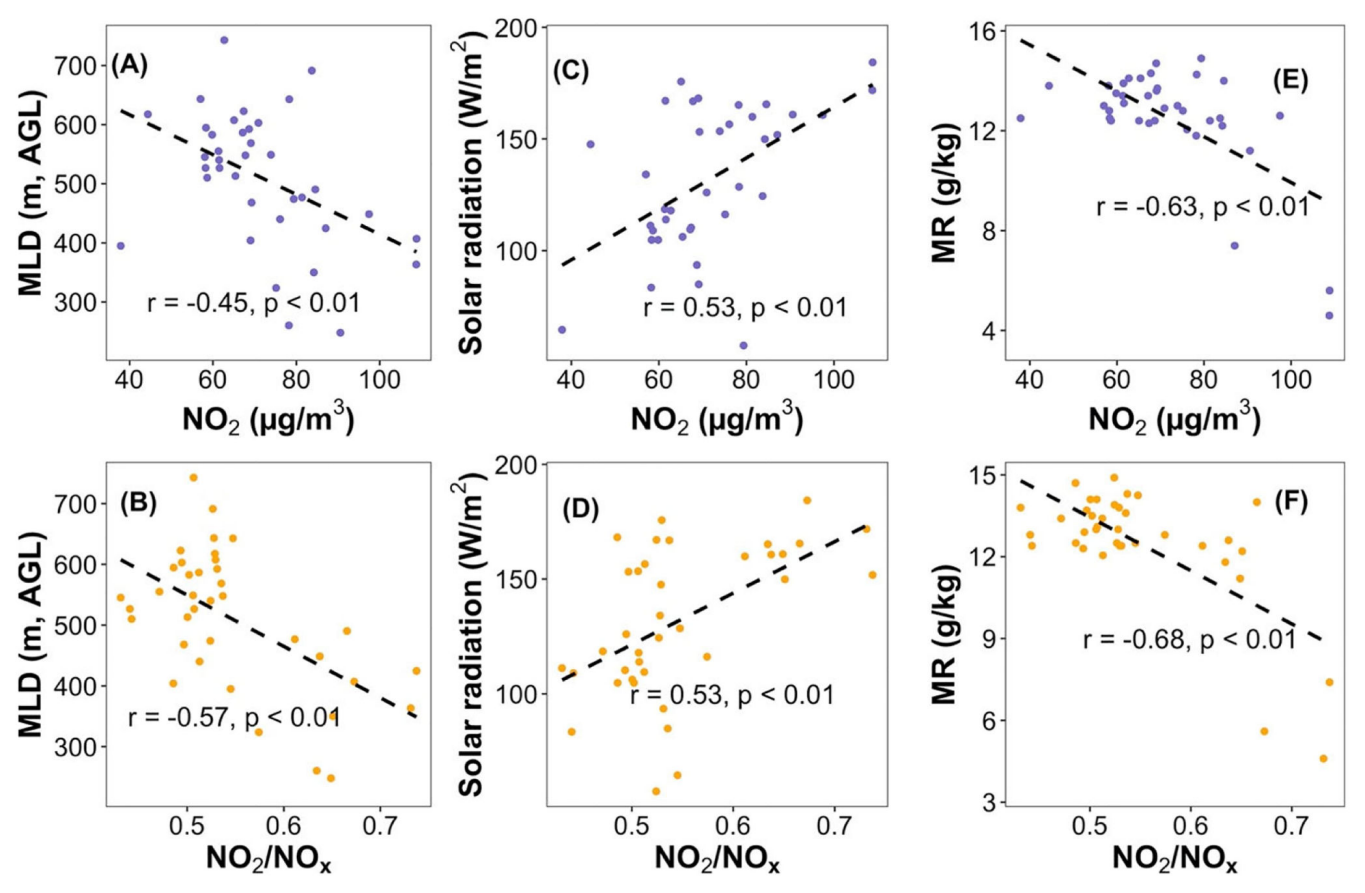
Relationship of weekly averaged mixing layer depth (A and B), incident solar radiation (C and D) and water vapor mixing ratio (MR) (E and F) with NO_2_ concentrations and NO_2_/NO_x_ ratios. The mixing layer depth, incident solar radiation and water vapor mixing ratio data were calculated through Hybrid Single-Particle Lagrangian Integrated Trajectory (HYSPLIT) 4 model (https://www.arl.noaa.gov/hysplit/hysplit/).

The GAMA experiences significant meteorological changes during the Harmattan season usually characterized by hotter, drier (higher temperature and lower relative humidity/water vapor mixing ratio) and stagnant wind that originates from the Saharan Desert (Figure S6). During this time, the mixing layer depth over the city lowers compared with non-Harmattan periods while incident solar radiation increases (Figure S7). We observed a fairly strong inverse relationship between the weekly averaged mixing layer depth and corresponding NO_2_ (*r* = −0.45, *p* < 0.01) and the NO_2_/NO_x_ ratio (*r* = −0.57, *p* < 0.01) (Figure 4A and 4B), pointing to likely enhancement of local pollutant concentrations due to slower vertical mixing during Harmattan.^20–22^ Also, we found a robust positive correlation between incident solar radiation and NO_2_ (*r* = 0.53, *p* < 0.01) and NO_2_/NO_x_ ratio (*r* = 0.53, *p* < 0.01) (Figure 4C and 4D), indicating higher photochemical activity (likely higher O_3_ concentration) during the Harmattan season.^22,39^ Further, we observed a strong inverse correlation of NO_2_ concentration (*r* = −0.63, *p* < 0.01) and NO_2_/NO_x_ ratio (*r* = −0.68, *p* < 0.01) with water vapor mixing ratio (Figure 4E and 4F), suggested that drier air promoted NO_2_ existence in the gas phase.

#### 3.2.4 Changes during COVID-19 pandemic lockdown

**Figure 5:**
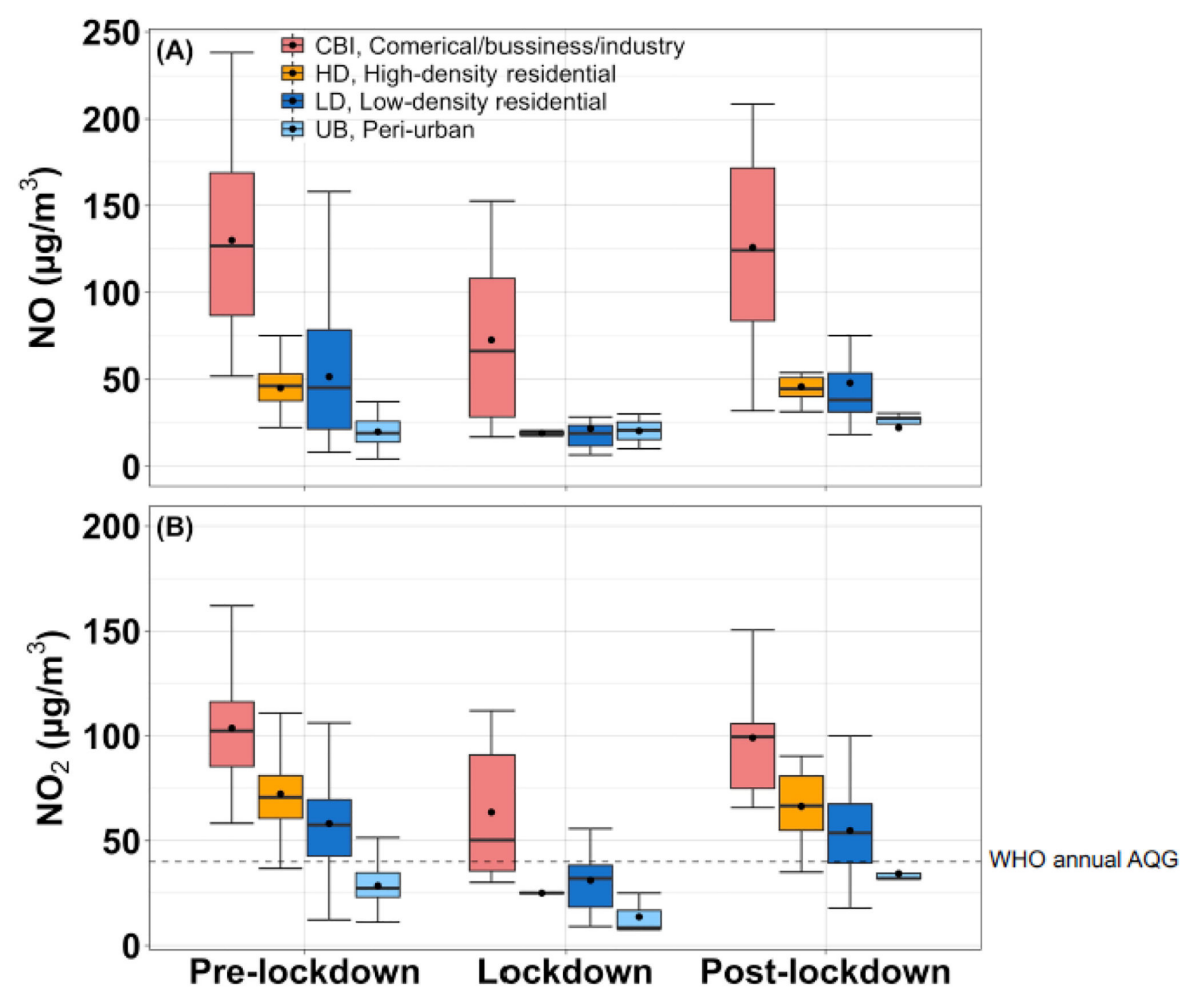
Distribution of pre-, during-, and post-COVID-19 lockdown (A) NO and (B) NO_2_ concentrations at the fixed (year-long) sites by site-type. The points in the box represent the mean values. Each box shows the median value (inside line), 25^th^ (lower), and 75th (upper) percentile of the data, and the lines extending from the boxes (whiskers) indicate variability outside the upper and lower quartiles. The dash line in (B) show the WHO guideline for annual NO_2_ concentrations (40 ug/m^3^).

COVID-19 pandemic lockdown of Accra had a considerable impact on NO_x_ and NO_2_ concentrations (Figure 5). The mean NO and NO_2_ concentrations during the lockdown both dropped to ~39 μg/m^3^, approximately 40% lower than the mean pre-lockdown levels (64 and 70 μg/m^3^, respectively); the levels rapidly returned to pre-lockdown concentrations in the post-lockdown period (70 and 68 μg/m^3^, respectively) (Table 2). Specifically, during the lockdown, NO and NO_2_ concentrations decreased the most at the residential sites (HD: 58% and 65%, respectively; and LD: 57% and 47%, respectively) than in the CBI areas (44% and 38%). The significant reduction in both NO and NO_2_ caused the mean NO_2_ levels in the residential neighborhoods to fall below the WHO 40 μg/m^3^ health guideline. The reductions appear consistent with Google Mobility report of 48-61% drop in visits to places like restaurants, markets and public transport hubs during the lockdown in Accra.^40^ Interestingly, NO_2_ at the UB background site also decreased by ~50% during the lockdown (14 μg/m^3^) in comparison to the pre- (29 μg/m^3^) and post-lockdown (34 μg/m^3^) levels, but with no significant change in NO levels in either of these periods (pre/during/post-lockdown ranged 20-22 μg/m^3^).

### 3.3 Correlations with traffic and biomass patterns

We combined data from both the fixed and rotating sites and tested the relative influence of traffic and biomass burning on NO_x_ and NO_2_ concentrations in the GAMA using a series of correlation analyses. We evaluated the levels in relation to distance of the measurement location to major roads (indicator for traffic) as well as with proportion of households using biomass fuel in the census enumeration area (EA) containing the measurement site (indicator for biomass burning). We caution, however, that biomass use data was derived from the 2010 national census and might not be an accurate reflection of the present community biomass use in these EAs. We caution further that there might be some level of correlation between traffic and biomass indicators. We found that both NO (*r* = −0.38, *p* < 0.01, Figure S9A) and NO_2_ (*r* = −0.55, *p* < 0.01, Figure S9C) levels decreased with distance from major roads. Concentrations measured at location within 500 m of a major road were significantly higher than those beyond 500 m (NO: 60 vs 33 μg/m^3^, *p* < 0.01; and NO_2_: 68 vs 41 μg/m^3^, *p* < 0.01). However, we observed no clear relationship between our samples and community biomass use based on the 2010 census (Figure S9E, S9F).

## 4 Discussion

In an expansive measurement campaign in one of SSA’s fast-growing metropolitan areas, we found that more than half of all sampling sites, including densely populated residential communities, had NO_2_ levels above the WHO annual guideline. The mean annual NO_2_ concentrations over the entire city, and in both CBI and residential neighborhoods also surpassed the WHO guideline. Levels were associated with indicators of road traffic and consistently high in the highly urbanized areas (especially in AMA and TMA), as well as in densely populated neighborhoods. We observed a strong seasonality in NO_2_ concentrations, most likely from the enhancement of local pollution during the harmattan due to changes in the local meteorology. These findings represent an increase of 53 – 152% over the last decade when compared to a small study conducted in two densely populated neighborhoods of Accra in 2006,^19^ which found low NO_2_ levels with virtually no variation across sites.

The current annual and seasonal mean NO_2_ concentrations in Accra are substantially higher than the mean Harmattan levels in Abidjan (Cote d’ivoire),^41^ annual mean in Cape Town (South Africa)^42^ and Dakar (Senegal),^43^ and non-Harmattan means in Bamako (Mali)^43^. Our findings could not be compared directly with regional estimates derived from satellite-based approaches,^44^ which provided only broad view of NO_2_ pollution in the sub-region but could not capture within-city spatial variability driven predominantly by local emission sources. Our results suggest that large within-city spatial variability exists in SSA cities, with levels in commercial areas and some residential communities several times higher than the peri-urban background areas. The mean annual NO_2_ levels in AMA are more than double those reported for major European cities, ^16,45^ New York (USA),^15^ and Beijing (China)^46^ (Figure S8). Overall, mean annual NO_x_ concentration in AMA is similar to concentrations during heavy polluted winter season in Beijing, China.^47,48^

NO_x_ is central to the formation of PM and ground level O_3_. In general, NO_x_ emissions from combustion sources are primarily in the form of NO, which further react with O_3_ to form NO_2_.^17^ Thus, NO/NO_x_ ratios are higher in direct/fresher emissions and lower in aged plumes.^49^ Our findings of variations in NO and NO_2_ levels by site-type and season indicate the important roles of fresh traffic emissions during the non-Harmattan period (i.e. higher NO) and enhanced secondary formation from both transboundary transport (emissions from open biomass burning) ^50^ and changes in local meteorology (amplification of local emissions) during the Harmattan period (i.e. higher NO_2_).^51^ We found no indication of increases in actual local emissions during the Harmattan season. But rather significant meteorological changes, including increased incident solar radiation (Figure S8) and temperature inversion during the Harmattan season,^21,22,26^ which could in turn increase regional production of O_3_ as observed by Marais *et al*. (2014) (in Nigeria) and Aghedo *et al*.(2007) (regional).^22,39^ Studies of PM concentrations in Accra have also reported elevated levels during Harmattan, but these increases have been mostly attributed to mineral dust transport from the Saharan Desert.^12,23,52^ Our findings in relation to mixing layer depth, incident solar radiation and water vapor mixing ratio during the Harmattan point to the important role of meteorology in amplifying local air pollution beyond just dust transport during the Harmattan.^21,22,53,54^

We found reductions in NO and NO_2_ concentrations during the COVID-19 lockdown of Accra, especially in CBI and residential (HD and LD) areas. This finding was supported by Google Mobility data for Accra, which also showed between 48-61% drop in mobility patterns for the same period. ^40^ This is another suggestive evidence of the significance of local traffic emissions and other household/community combustion related activities on NO_x_ pollution in Accra. At our UB sites, which were expected to be less influenced by direct emissions, we observed little changes in NO levels in pre-, during-, and post-lockdown periods, contrary to substantial decrease in NO_2_ levels during the lockdown, signifying broader impact of the lockdown through reduced secondary formation of pollutants from local emissions.

A recent paper found reductions in ambient PM_2.5_ pollution in Accra when compared to 2006/2007 data. ^25^ The paper noted that concentrations in the city have plateaued at levels lower than those seen in large Asian megacities. Combined with our analysis, there is a strong evidence that air pollution levels in Accra can be reduced city-wide if necessary policies are implemented. Like observed globally during the COVID-19 pandemic when reductions in transportation sector emissions accounted for about 31-60% reductions in NO_2_ levels, policies targeted at reducing traffic emissions in Accra would greatly improve air quality in the city. Specifically, with the rapid growth of vehicle numbers, policies on traffic (congestion) control and better road network planning, especially in relation to residential areas, are urgently needed. Additionally, Ghana’s efforts in reducing air pollution, including promotion of liquefied petroleum gas for household use, adoption of low sulfur content standard in diesel, and adoption of Euro 4/IV emission standards would require stronger enforcement to ensure cleaner air for all.

### 4.1 Strength and limitation

The main strength of our paper is its large scope and setting, a place where local data, evidence, and capacity building in this context are needed. We implemented the most comprehensive city-wide field campaign spanning a wide spatial and temporal extent than in any SSA city. We also combined geo-referenced data to assess impact of different emission sources, including traffic, biomass use, and meteorology, on ambient NO and NO_2_ concentrations. We were able to document substantial increase in the levels over a decade as well as the impact of Covid-19 lockdown on local emission in the city.

There are several limitations to our study. First, we could not compare our data with reference NO_x_ monitors. Although no chemiluminescence measurements are conducted in Accra, Ogawa samplers have been comprehensively characterized, including in SSA setting and shown to be consistent.^35,36,55,56^ Second, due to Covid-19 pandemic, we had some gaps in our data. However, we still collected enough data over the entire year to provide large scale overview of temporal patterns of NO and NO_2_ pollution in Accra. Third, we had no data on O_3_, which could provide additional insights into the spatial distribution of NO and NO_2_. In evaluating the potential impact of biomass on NO_2_ and NO_x_ in the city, we relied on 2010 census data because the 2020 national population census was still ongoing at the time of our study. The 2010 data likely did not accurately reflect the current household biomass use in the city as there were indications of a decline since 2010. This may have influenced our assessment of the role of biomass burning on the NO and NO_2_ emissions. Further, although the HYSPLIT model is well established and commonly used in a lot of studies, we did not have direct measurements (such as radiosonde profiles, vertical meteorology profiles, etc.) in Accra to determine mixing layer depth. Lastly, while the passive Ogawa is a cost-effective option in SSA settings where electricity from the grid to run active samplers is unreliable, we could not assess the levels at finer resolutions (e.g. diurnal patterns).

## 5 Conclusion

Ambient NO_x_ levels in Accra are rising and NO_2_ concentrations are now significantly higher than international health guidelines, especially in CBI and densely populated residential neighborhoods which are dominated by road traffic. With the expectation of further increases in road traffic congestion due to urban population growth, air pollution in Accra (and in other SSA cites) will likely be dominated by road traffic emissions. Meteorological changes during the Harmattan also appear to enhance local NO_2_ levels in Accra. We recommend an integrated air quality management approach with emphasis on sources, land use, and meteorology to address growing urban air pollution problems in Accra and elsewhere in the sub-region.

## Supplementary Material

Appendix

## Figures and Tables

**Table 1:** Weekly integrated NO, NO_2_, NO_x_ concentrations (μg/m^3^), and NO/NO_x_ ratio at all rotating sites by site type.

City region	Measureme nt site		NO		NO_2_		NO_x_		NO/NO_x_	
	Type	n	Mean (SD)	Range	Mean (SD)	Range	Mean (SD)	Range	Mean (SD)	Range
GAMA	All sites	124	38 (26)	6 – 169	48 (26)	9 – 139	82 (45)	29 – 297	0.46 (0.10)	0.19 – 0.79
	CBI	23	66 (40)	25 – 169	76 (25)	46 – 139	134 (61)	81 – 297	0.47 (0.09)	0.31 – 0.57
	HD	32	44 (22)	19 – 110	56 (21)	20 – 93	100 (38)	41 – 180	0.44 (0.07)	0.33 – 0.63
	LD	42	33 (19)	12 – 94	43 (18)	10 – 100	73 (33)	30 – 173	0.45 (0.11)	0.28 – 0.79
	UB	27	23 (5)	6 – 30	24 (11)	9 – 62	48 (12)	29 – 82	0.49 (0.12)	0.19 – 0.71
AMA	All sites	44	47 (30)	19 – 131	64 (26)	20 – 139	102 (48)	41 – 232	0.44 (0.12)	0.28 – 0.79
TMA	All sites	16	44 (22)	24 – 92	51 (15)	28 – 89	96 (35)	53 – 182	0.45 (0.07)	0.33 – 0.57
Other *	All sites	64	31 (22)	6 – 169	36 (21)	9 – 126	67 (41)	29 – 297	0.47 (0.10)	0.19 – 0.71

* Other municipalities in the GAMA beside AMA and TMA.

**Table 2:** Weekly integrated NO, NO_2_, NO_x_ concentrations (μg/m^3^), and NO/NO_x_ ratio at the fixed (year-long) sites by site type and season.

Site type	Period	NO		NO_2_		NO_x_		NO/NO_x_	
		Mean (SD)	Range	Mean (SD)	Range	Mean (SD)	Range	Mean (SD)	Range
**All** **sites** **(n = 10)**	**Annual**	**68 (52)**	**0.4 – 238**	**70 (33)**	**8 – 199**	**139 (77)**	**16 – 359**	**0.46 (0.14)**	**0.01 – 0.84**
	Harmattan	59 (41)	4 – 165	87 (35)	19 – 199	147 (69)	43 – 333	0.37 (0.13)	0.1 – 0.66
	Non-Harmattan	72 (56)	0.4 – 238	63 (29)	8 – 150	136 (81)	16 – 359	0.49 (0.12)	0.01 – 0.84
	Pre-lockdown	70 (52)	4 – 238	72 (32)	11 – 199	144 (76)	28 – 354	0.45 (0.14)	0.10 – 0.84
	Covid-19 lockdown	39 (41)	6.7 – 153	39 (29)	7.5 – 112	78 (68)	16 – 265	0.47 (0.10)	0.30 – 0.71
	Post-lockdown	68 (51)	0.4 – 208	68 (32)	18 – 150	137 (80)	36 – 358	0.47 (0.11)	0.01 – 0.65
CBI (n = 3)	**Annual**	**126 (50)**	**17 – 238**	**101 (28)**	**30 – 199**	**228 (62)**	**48 – 359**	**0.54 (0.11)**	**0.24 – 0.78**
	Harmattan	107 (33)	52 – 165	123 (25)	90 – 199	229 (40)	176 – 333	0.46 (0.09)	0.24 – 0.64
	Non-Harmattan	134 (54)	17 – 238	92 (23)	30 – 150	228 (70)	48 – 359	0.57 (0.09)	0.32 – 0.78
	Pre-lockdown	130 (47)	52 – 238	104 (24)	58 – 199	236 (51)	151 – 354	0.55 (0.11)	0.24 – 0.78
	Covid-19 lockdown	73 (54)	17 – 153	64 (34)	30 – 112	136 (86)	48 – 265	0.50 (0.09)	0.35 – 0.6
	Post-lockdown	126 (55)	32 – 208	99 (28)	66 – 150	225 (78)	101 – 359	0.54 (0.1)	0.32 – 0.65
HD (n = 2)	**Annual**	**45 (12)**	**13 – 75**	**70 (18)**	**25 – 127**	**116 (21)**	**43 – 165**	**0.39 (0.09)**	**0.14 – 0.67**
	Harmattan	38 (11)	13 – 56	85 (16)	57 – 127	123 (20)	94 – 165	0.31 (0.07)	0.14 – 0.46
	Non-Harmattan	46 (11)	18 – 75	64 (16)	25 – 97	113 (21)	43 – 160	0.42 (0.07)	0.19 – 0.67
	Pre-lockdown	45 (12)	13 – 75	72 (16)	37 – 127	119 (15)	94 – 165	0.38 (0.1)	0.14 – 0.67
	Covid-19 lockdown	19 (2)	18 – 21	25 (0.6)	25 – 25	44 (2)	43 – 45	0.43 (0.03)	0.41 – 0.46
	Post-lockdown	46 (11)	32 – 69	66 (18)	35 – 90	112 (28)	67 – 160	0.41 (0.04)	0.35 – 0.47
LD (n = 4)	**Annual**	**49 (32)**	**7 – 158**	**56 (25)**	**9 – 132**	**106 (50)**	**16 – 221**	**0.44 (0.14)**	**0.11 – 0.84**
	Harmattan	47 (29)	8 – 108	75 (27)	27 – 132	122 (50)	56 – 221	0.36 (0.12)	0.11 – 0.56
	Non-Harmattan	51 (34)	7 – 158	49 (20)	9 – 100	100 (48)	16 – 206	0.48 (0.13)	0.12 – 0.84
	Pre-lockdown	51 (33)	8 – 158	58 (25)	12 – 132	111 (50)	28 – 221	0.44 (0.15)	0.11 – 0.84
	Covid-19 lockdown	22 (16)	7 – 57	31 (16)	9 – 56	53 (31)	16 – 113	0.40 (0.07)	0.3 – 0.51
	Post-lockdown	48 (26)	18 – 110	55 (23)	18 – 100	103 (47)	36 – 206	0.46 (0.07)	0.36 – 0.59
UB (n = 1)	**Annual**	**20 (9)**	**0.4 – 37**	**28 (10)**	**8 – 54**	**49 (9)**	**18 – 70**	**0.42 (0.17)**	**0.01 – 0.74**
	Harmattan	16 (11)	4 – 37	35 (9)	19 – 52	51 (6)	43 – 60	0.31 (0.18)	0.1 – 0.66
	Non-Harmattan	22 (8)	0.4 – 37	26 (10)	8 – 54	48 (11)	18 – 70	0.46 (0.15)	0.01 – 0.74
	Pre-lockdown	20 (9)	4 – 37	29 (9)	11 – 52	49 (7)	38 – 70	0.40 (0.16)	0.1 – 0.74
	Covid-19 lockdown	20 (10)	10 – 30	14 (10)	8 – 25	34 (19)	18 – 55	0.61 (0.09)	0.55 – 0.71
	Post-lockdown	22 (12)	0 – 31	34 (12)	20 – 54	57 (6)	48 – 63	0.39 (0.22)	0.01 – 0.59

## Data Availability

The measurement data that support the findings of this study are available upon request from the authors.
