# Supplementary material for: Nitrogen oxides (NO and NO2) pollution in the Accra metropolis: Spatiotemporal patterns and the role of meteorology
Source: Sci Total Environ. Author manuscript; Available in PMC 2021 Sep 14. (PMC7611659; doi:10.1016/j.scitotenv.2021.149931)
Supplement: Appendix [file EMS134135-supplement-Appendix.pdf]

Appendix A. Supplementary data

Nitrogen oxides (NO and NO_2) pollution in the Accra metropolis: Spatiotemporal patterns and the role of meteorology

Jiayuan Wang[1], Abosede Sarah Alli[1], Sierra Clark[2], Allison Hughes[3], Majid Ezzati[2,4,5,6], Andrew Beddows[7], Jose Vallarino[8], James Nimo[3], Josephine Bedford-Moses[3], Solomon Baah[3], George Owusu[9], Ernest Agyemang[10], Frank Kelly[4,7], Benjamin Barratt[4,7], Sean Beevers[4], Samuel Agyei-Mensah[10], Jill Baumgartner[11,12], Michael Brauer[13], Raphael E Arku[1*]

[1]Department of Environmental Health Sciences, School of Public Health and Health Sciences, University of Massachusetts, Amherst, USA
[2]Department of Epidemiology and Biostatistics, School of Public Health, Imperial College, London, UK
[3]Department of Physics, University of Ghana, Legon, Ghana
[4]MRC Centre for Environment and Health, Imperial College London, London, UK
[5]Abdul Latif Jameel Institute for Disease and Emergency Analytics, Imperial College London, London, UK
[6]Regional Institute for Population Studies, University of Ghana, Accra, Ghana
[7]NIHR HPRU in Environmental Exposures and Health, Imperial College London, UK
[8]Harvard T.H. Chan School of Public Health, Boston, MA, USA
[9]Insitute of Statistical, Social and Economic Research, University of Ghana, Legon, Ghana
[10]Department of Geography and Resource Development, University of Ghana, Legon, Ghana
[11]Institute for Health and Social Policy, McGill University, Montreal, Canada
[12]Department of Epidemiology, Biostatistics, and Occupational Health, McGill University, Montreal, Canada
[13]School of Population and Public Health, The University of British Columbia, Vancouver, Canada

*Correspondence to:
Raphael E Arku
School of Public Health and Health Sciences
University of Massachusetts Amherst, MA, USA
E-mail: rarku@umass.edu

*** Color does not need to be used for any figures in print.

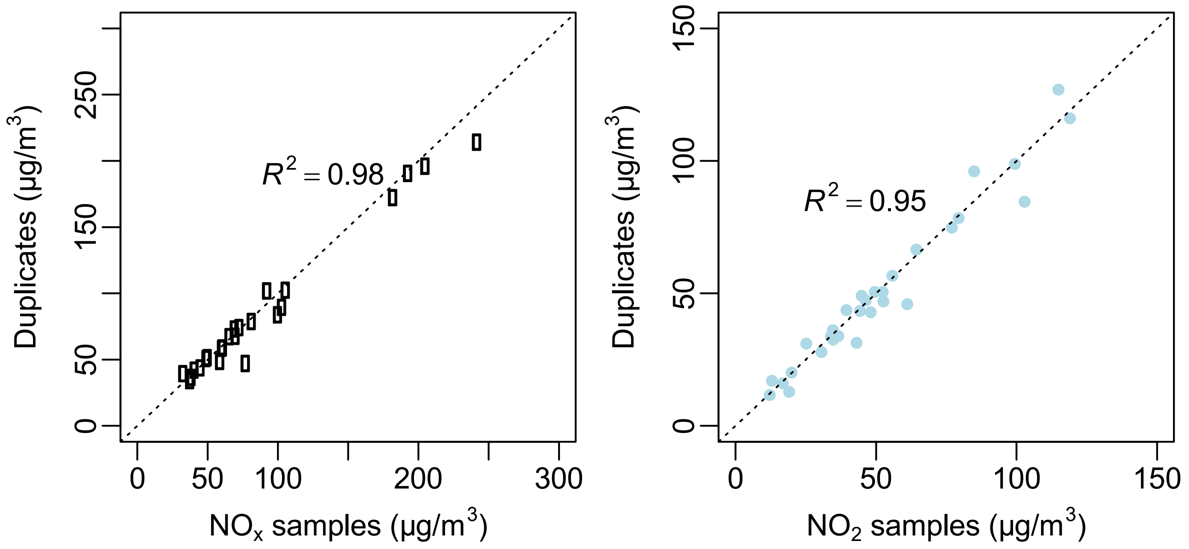

Figure S1. Correlation test between samples and duplicates.

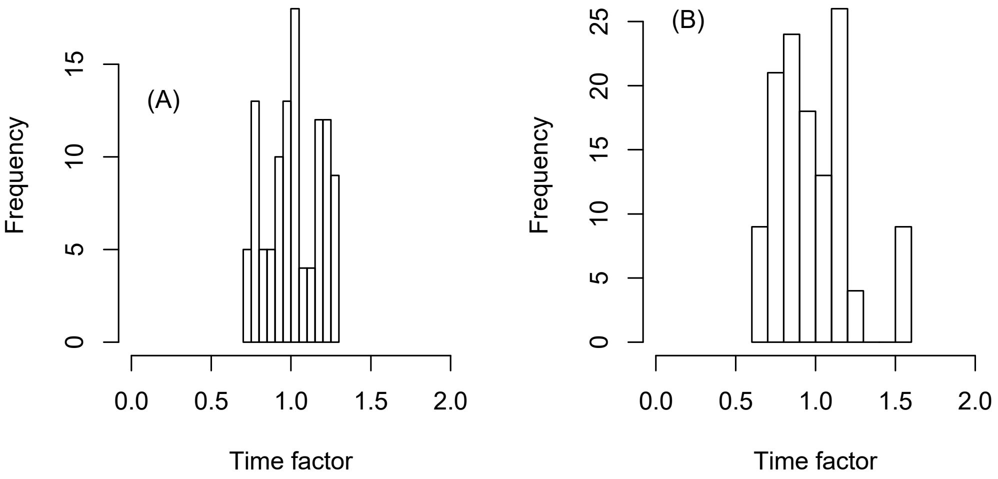

Figure S2. Histogram of time correction factor of NO (A) and NO_2 (B) for temporal adjustment.

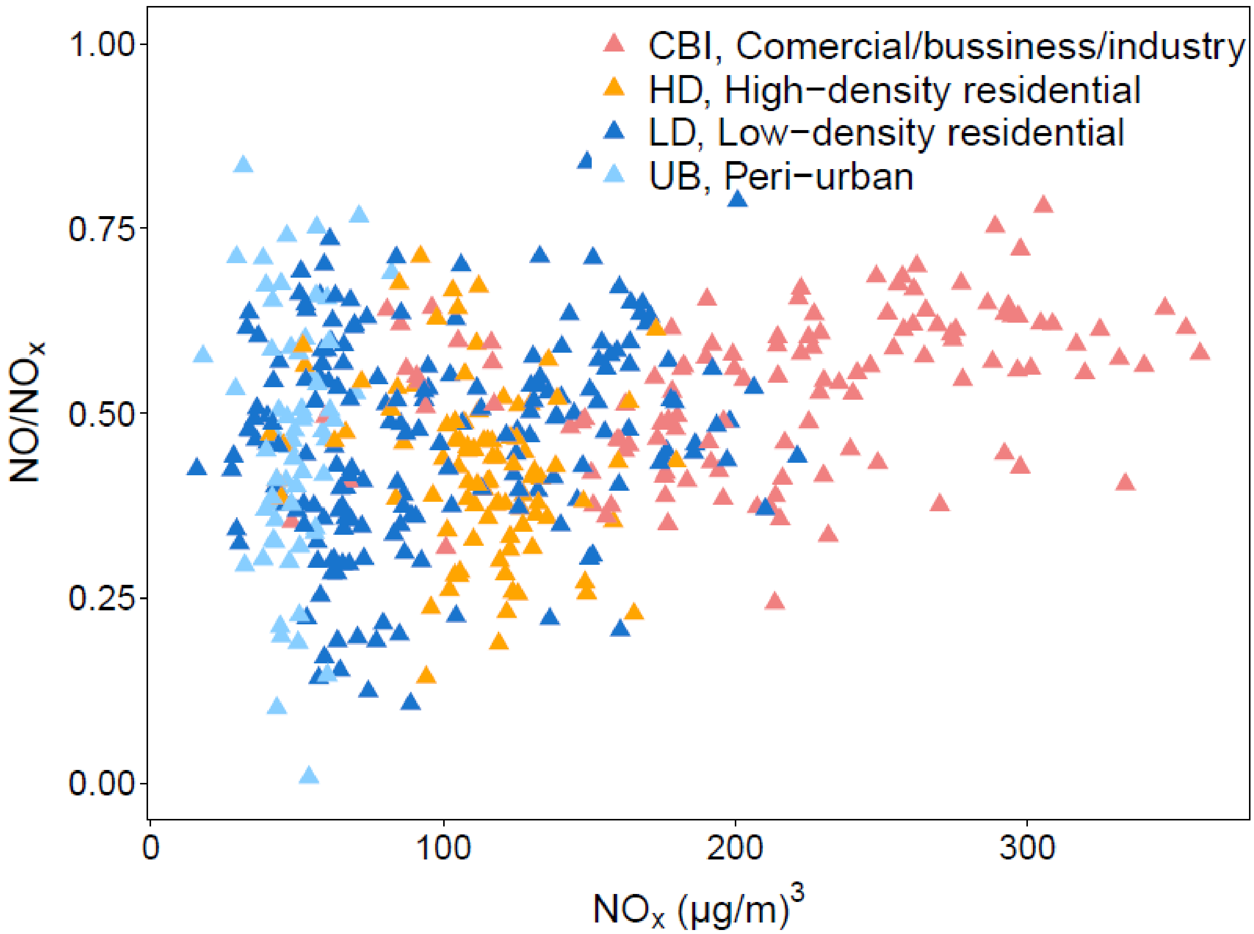

Figure S3. Scatter plot of NO/NO_x ratio with NO_x concentrations.

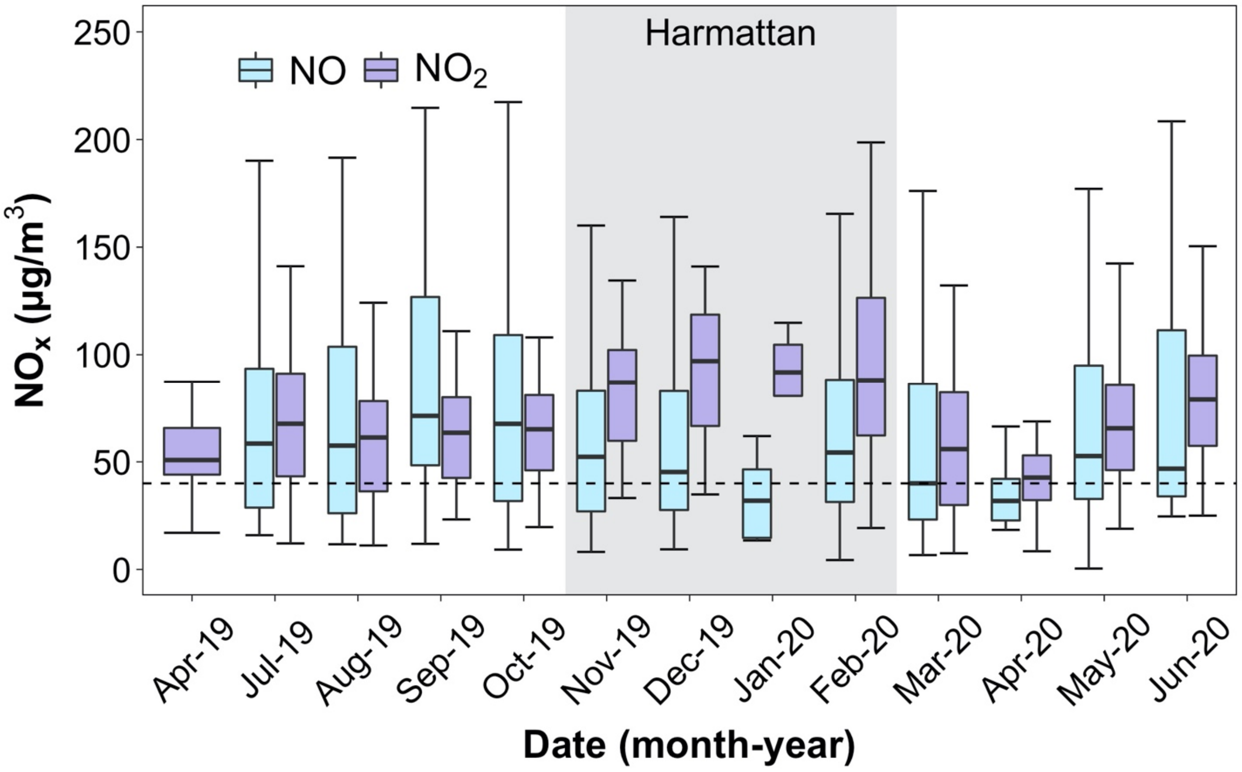

Figure S4. Monthly average concentrations of NO and NO_2 at all fixed sites. The dash line indicate the WHO annual guideline of 40 µg/m^3 for NO_2. Here, we included pilot NO_2 data collected in April.

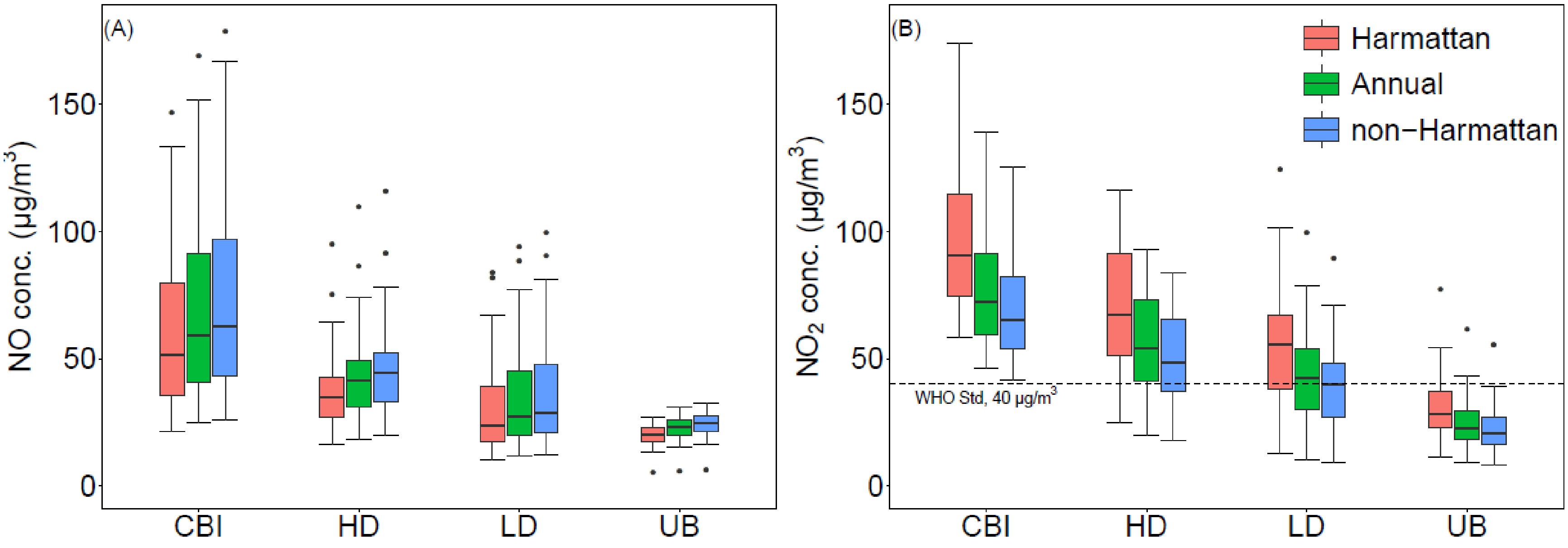

Figure S5. Annual and seasonal mean NO_2 and NO concentrations by site-types: commercial/business/industry (CBI), high-density residential (HD), low-density residential (LD) and urban background (UB) sites. The input data represent seasonal and annual mean equivalents for all monitoring sites.

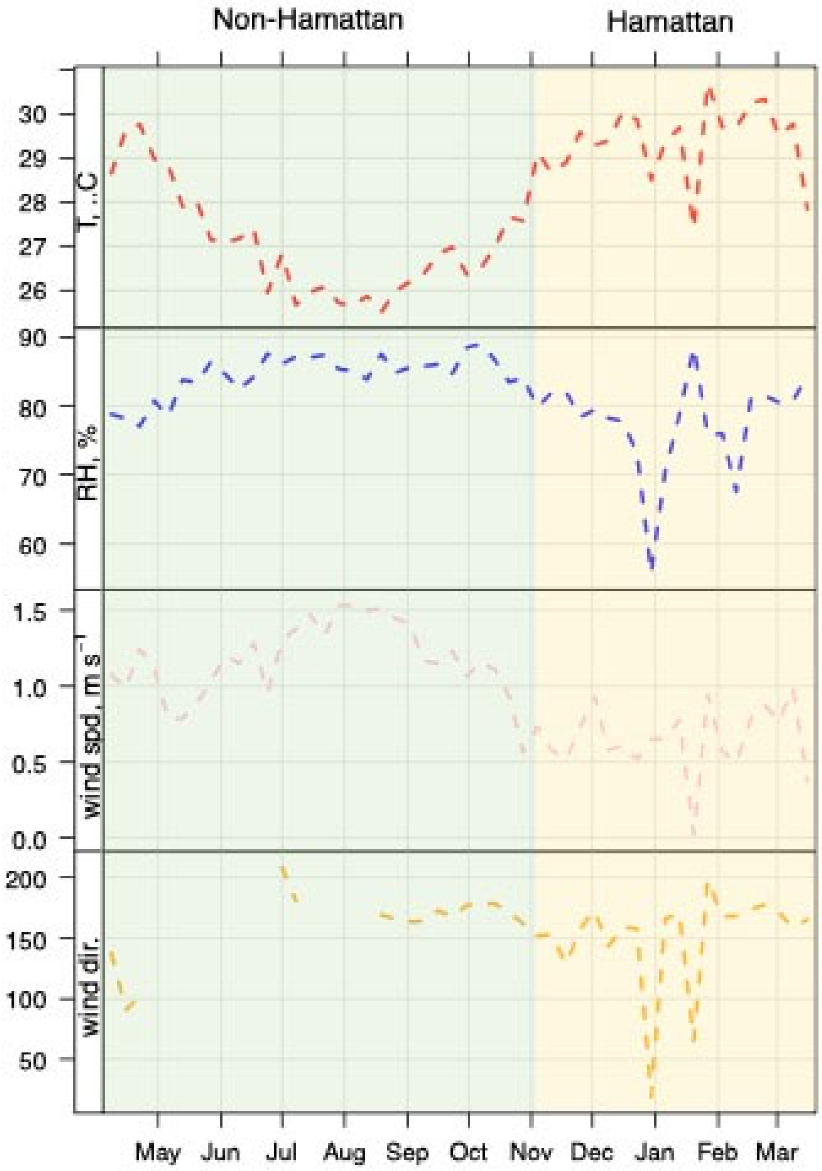

Figure S6. Time series of meteorological parameters (temperature, relative humidity, wind speed and wind direction) in Accra from April 2019 to March 2020. The light green shade covers non-Harmattan period, and the light orange shade covers Harmattan period.

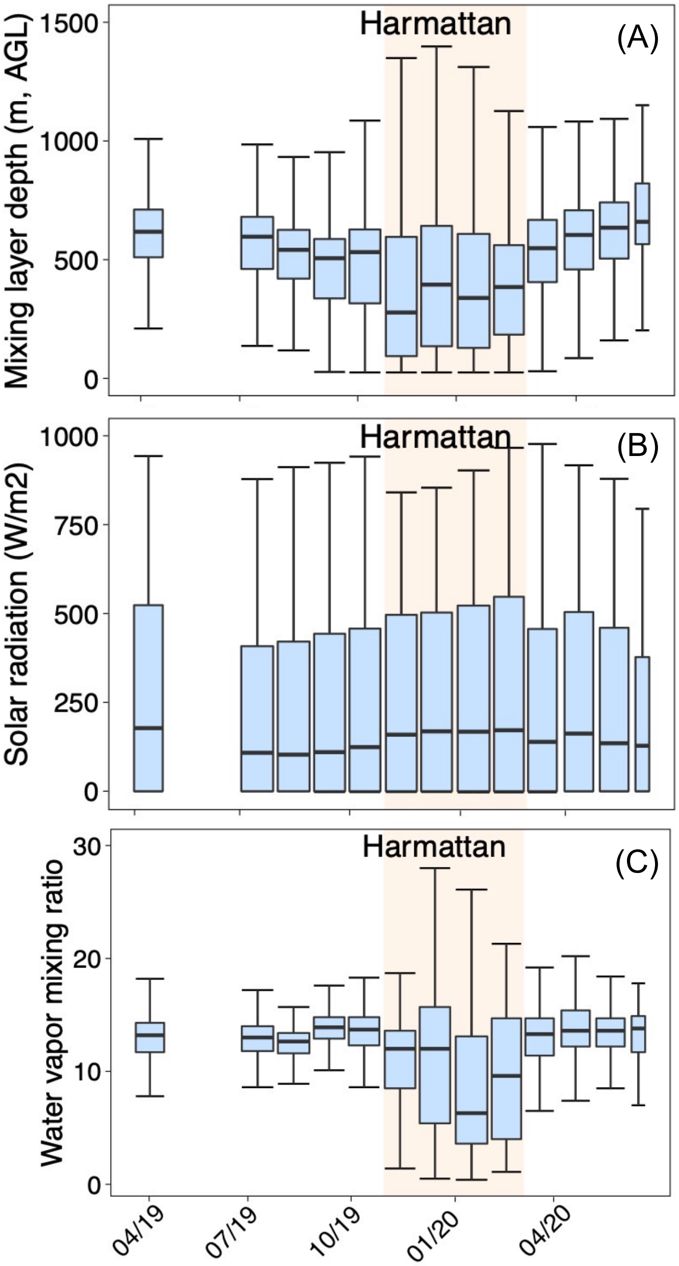

Figure S7. Monthly mixing layer depth (m, above ground level) (A) Incident solar radiation, and (B) Water vapor mixing ratio (C) during the full campaign period. The line in the box represents the median.

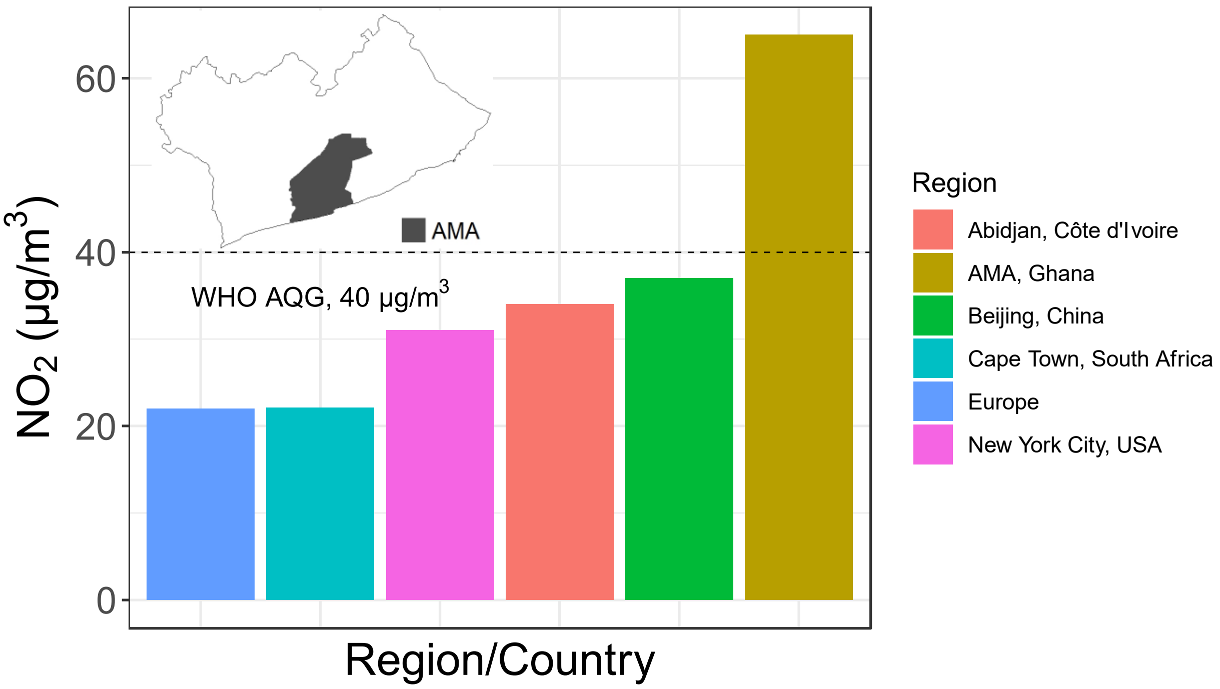

Figure S8. Comparison of annual mean NO_2 concentrations in Accra Metropolitan Area (AMA) and other cities/regions in the world. The dash line is the WHO guideline for annual concentrations of NO_2 (40 ug/m^3).

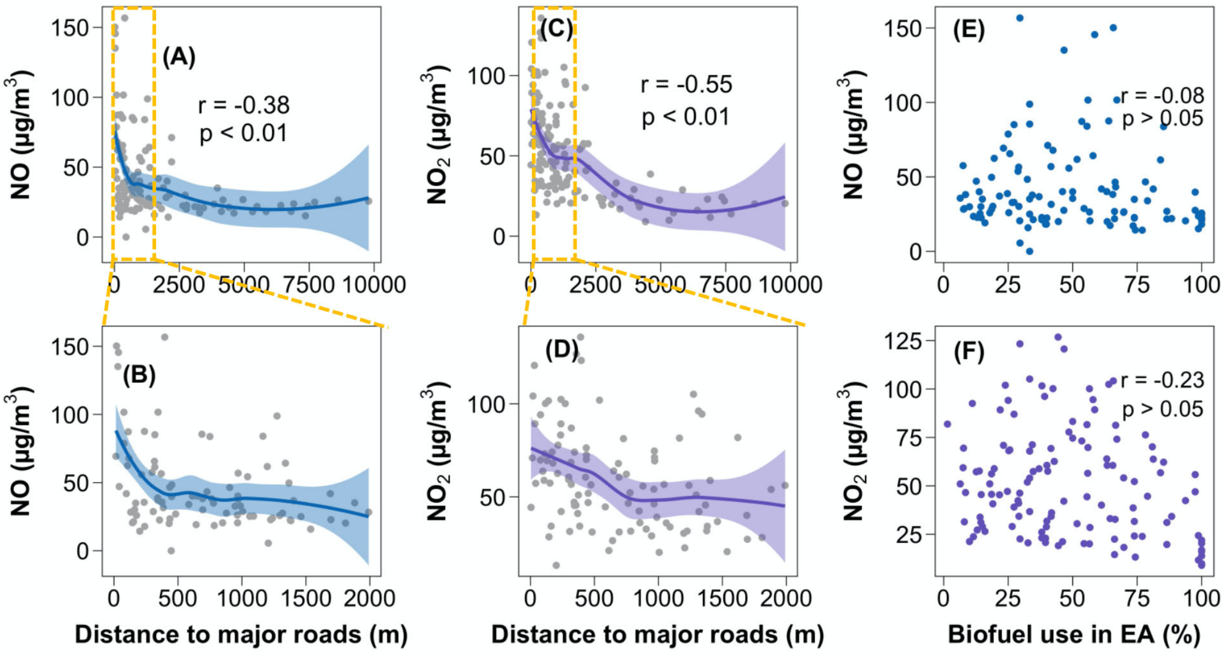

Figure S9. Relationship between NO_x concentrations and (A ~D) distance to major roads, and (E~F) biomass use percentage in enumeration area (EA) containing the monitoring locations. The smooth trend method is loess, and the shade areas are the standard errors. The road network data was downloaded from OpenStreetMap (2019). We caution that the 2010 census biomass use data may not reflect present usage (Ghana Statistic Service, 2010).
